# Supplementary material for: Design, Synthesis, and Biological Evaluation of N-Acylhydrazones and Their Activity Against Leishmania amazonensis Promastigotes
Source: Microorganisms. 2025 Jul 2;13(7):1563. doi: 10.3390/microorganisms13071563 (PMC12298860; doi:10.3390/microorganisms13071563)
Supplement: Supplementary file 1 [file microorganisms-13-01563-s001.zip › RMN amostras-20241114T155656Z-001.zip!/RMN amostras/composto 14 - PMM 105-17 1H.pdf]

PMM 105-17 - Joao Mafra
Op. Eliane

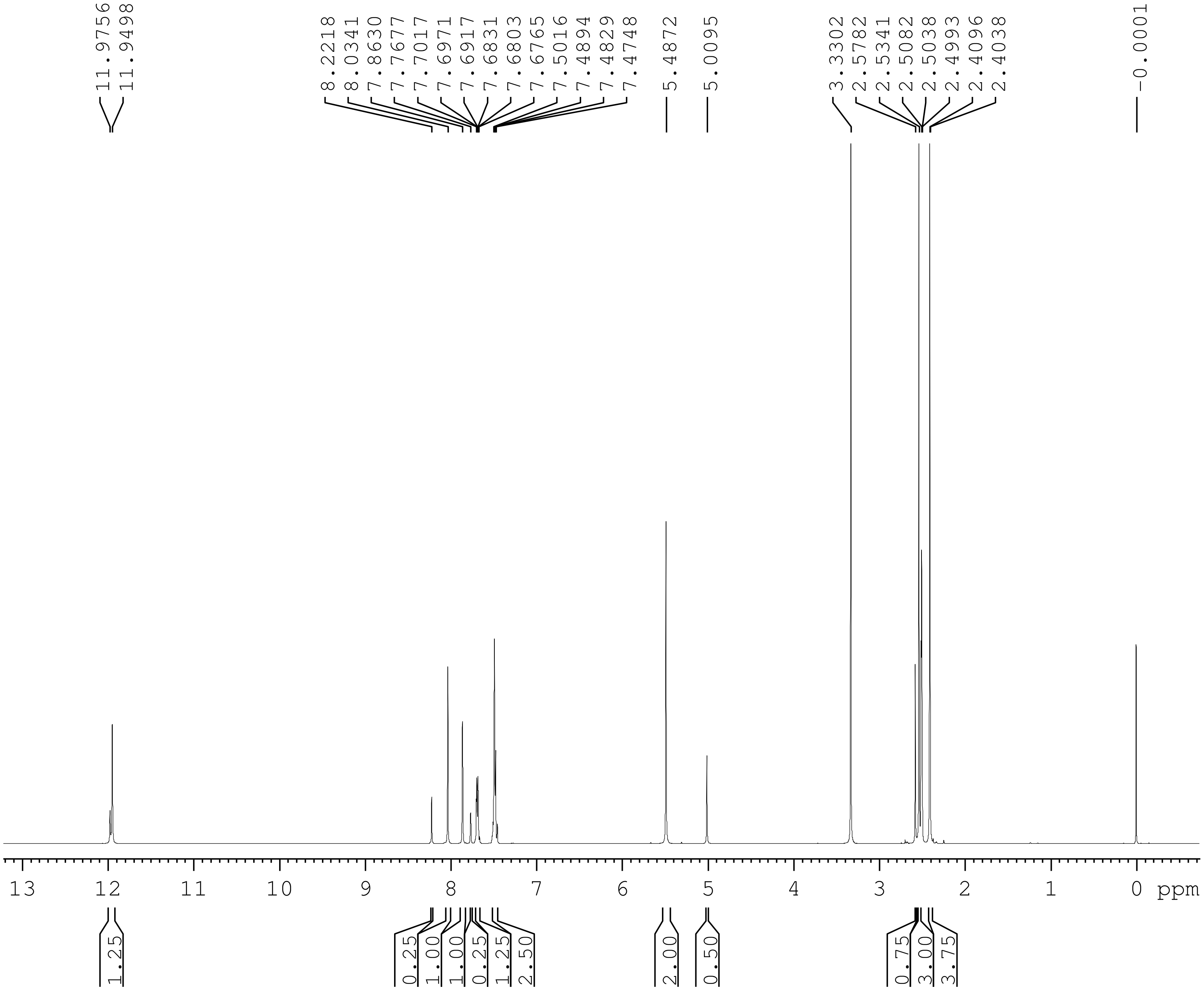

BRUKER

Current Data Parameters		
NAME	si103181832	
EXPNO	10	
PROCNO	1	
F2 - Acquisition Parameters		
Date_	20180320	
Time	11.17	h
INSTRUM	spect	
PROBHD	Z122623_0041 (	
PULPROG	zg30	
TD	65536	
SOLVENT	DMSO	
NS	32	
DS	2	
SWH	8012.820	Hz
FIDRES	0.244532	Hz
AQ	4.0894465	sec
RG	132.74	
DW	62.400	usec
DE	10.00	usec
TE	298.0	K
D1	1.00000000	sec
TD0	1	
SFO1	400.1524711	MHz
NUC1	1H	
P1	11.50	usec
PLW1	7.30000019	W
F2 - Processing parameters		
SI	65536	
SF	400.1500015	MHz
WDW	EM	
SSB	0	
LB	0.30	Hz
GB	0	
PC	1.00	
